# Unraveling the MRI‐Based Microstructural Signatures Behind Primary Progressive and Relapsing–Remitting Multiple Sclerosis Phenotypes

**DOI:** 10.1002/jmri.27806

**Published:** 2021-06-30

**Authors:** Ilaria Boscolo Galazzo, Lorenza Brusini, Muge Akinci, Federica Cruciani, Marco Pitteri, Stefano Ziccardi, Albulena Bajrami, Marco Castellaro, Ahmed M.A. Salih, Francesca B. Pizzini, Jorge Jovicich, Massimiliano Calabrese, Gloria Menegaz

**Affiliations:** ^1^ Department of Computer Science University of Verona Verona Italy; ^2^ Center for Mind/Brain Sciences University of Trento Trento Italy; ^3^ Neurology Unit, Department of Neurosciences, Biomedicine and Movement Sciences University of Verona Verona Italy; ^4^ Radiology Unit, Department of Diagnostic and Public Health University of Verona Verona Italy

**Keywords:** diffusion MRI, 3D‐SHORE, morphometry, gray matter, EDSS, SVM

## Abstract

**Background:**

The mechanisms driving primary progressive and relapsing–remitting multiple sclerosis (PPMS/RRMS) phenotypes are unknown. Magnetic resonance imaging (MRI) studies support the involvement of gray matter (GM) in the degeneration, highlighting its damage as an early feature of both phenotypes. However, the role of GM microstructure is unclear, calling for new methods for its decryption.

**Purpose:**

To investigate the morphometric and microstructural GM differences between PPMS and RRMS to characterize GM tissue degeneration using MRI.

**Study Type:**

Prospective cross‐sectional study.

**Subjects:**

Forty‐five PPMS (26 females) and 45 RRMS (32 females) patients.

**Field Strength/Sequence:**

3T scanner. Three‐dimensional (3D) fast field echo T1‐weighted (T1‐w), 3D turbo spin echo (TSE) T2‐w, 3D TSE fluid‐attenuated inversion recovery, and spin echo‐echo planar imaging diffusion MRI (dMRI).

**Assessment:**

T1‐w and dMRI data were employed for providing information about morphometric and microstructural features, respectively. For dMRI, both diffusion tensor imaging and 3D simple harmonics oscillator based reconstruction and estimation models were used for feature extraction from a predefined set of regions. A support vector machine (SVM) was used to perform patients' classification relying on all these measures.

**Statistical Tests:**

Differences between MS phenotypes were investigated using the analysis of covariance and statistical tests (*P* < 0.05 was considered statistically significant).

**Results:**

All the dMRI indices showed significant microstructural alterations between the considered MS phenotypes, for example, the mode and the median of the return to the plane probability in the hippocampus. Conversely, thalamic volume was the only morphometric feature significantly different between the two MS groups. Ten of the 12 features retained by the selection process as discriminative across the two MS groups regarded the hippocampus. The SVM classifier using these selected features reached an accuracy of 70% and a precision of 69%.

**Data Conclusion:**

We provided evidence in support of the ability of dMRI to discriminate between PPMS and RRMS, as well as highlight the central role of the hippocampus.

**Level of Evidence:**

2

**Technical Efficacy Stage:**

3

Multiple sclerosis (MS) is an inflammatory‐neurodegenerative disease of the central nervous system (CNS) characterized by demyelinating lesions and axonal loss in both the brain and the spinal cord, leading to severe symptoms such as loss in physical function, fatigue, and cognitive decline.^1^ Several major MS types have been identified so far, among which primary progressive MS (PPMS) and relapsing–remitting MS (RRMS) are the two most common forms.^2^ While the PPMS form is characterized by progressive worsening with minor or no remissions, RRMS is characterized by acute inflammatory attacks followed by remission.^2^, ^3^ Demyelinating lesions and regional brain atrophy are generally observed in both cases, but recent studies provided evidence of the existence of distinct patterns of demyelination in these two forms,^4^ suggesting the presence of different subserving mechanisms, albeit still largely unclear.^3^

During the last decades, non‐invasive neuroimaging techniques have been increasingly exploited to shed light on the multiple changes induced by MS disease.^5^ In particular, while MS has traditionally been considered as a chronic inflammatory‐demyelinating condition affecting almost exclusively the white matter (WM), pathology studies have shown an extensive involvement of the gray matter (GM) in different forms of MS, as confirmed by several magnetic resonance imaging (MRI) studies detecting both demyelination and atrophy in cortical and deep GM structures.^6^

In this context, valuable information can be derived from diffusion MRI (dMRI) which allows to quantify, in vivo, the microstructural properties of brain tissues.^7^ In recent years, different models have been proposed to numerically describe microstructural properties. Among the several taxonomies proposed in the literature, we refer to the two main classes as compartmental models and analytical models, respectively.^8^

Compartmental models rely on predefined assumptions on the biophysical properties of the tissue, while analytical models look at the diffusion signal as a function that can be represented on a given basis. Among the compartmental models, NODDI (neurite orientation dispersion and density imaging) and CHARMED (composite hindered and restricted model of diffusion) are the most widespread, especially for clinical applications.^9^, ^10^ However, these models rely on strict assumptions about the tissue microstructure that bias their descriptive power and have strong implications on the interpretability as well as on the specificity of the results.^11^ On the other hand, signal models are based solely on the analytical representation of the dMRI signal in a given basis of functions, making no assumptions on the tissue structure.^8^, ^12^ As a consequence, signal representations can be applied to any condition and tissue type, providing an indirect measure of the tissue properties through the coefficients of the series expansion from which microstructural descriptors are derived.^13^ For a complete overview of these methods, we refer to some recent reviews on the topic.^7^, ^8^

In light of these considerations, our study focused on analytical signal models and, specifically, on those that rely on the estimation of the ensemble average propagator (EAP).^12^, ^13^ This function characterizes the restricted displacement of water molecules as defined by the local microscopic properties of brain tissues. Among these, diffusion tensor imaging (DTI) is the simplest and is widely used in clinical practice.^14^ Nonetheless, DTI assumes a Gaussian model for the diffusion process and, therefore, its applicability is precluded in brain areas where this assumption fails, such as in those brain regions having complex structure (e.g. WM fibers crossings). The simple harmonic oscillator‐based reconstruction and estimation (SHORE) model allows overcoming these limitations.^13^ Several indices can be derived in the analytical form, which have proved to be effective for characterizing brain tissues in vivo^15^ in healthy subjects as well as in pathological populations.^16^ The three‐dimensional (3D)‐SHORE model has demonstrated good performance in probing stroke‐induced microstructural modulations occurring over several GM areas.^17^ Moreover, in some recent studies it has been shown to be sensitive to GM alterations pointing to different MS stages and to be associated with cognitive impairment.^18^

Altered diffusivity and anisotropy patterns in the normal‐appearing GM have been detected in several DTI‐based studies on RRMS, PPMS, and secondary progressive MS patients.^19^, ^20^ In this respect, advanced models have revealed an increased specificity and sensitivity to neurodegeneration when compared to conventional DTI derived measurements. In particular, Granberg et al^21^ explored the NODDI model for the characterization of normal‐appearing WM and GM. They found focal abnormalities in the cortex and more diffusively in the WM in the early stages of MS. In addition, De Santis et al^22^ investigated several dMRI biomarkers from basic (DTI) to advanced (NODDI, CHARMED, and diffusion kurtosis imaging) models, though in a small cohort of MS patients compared to controls. They demonstrated that advanced models have increased specificity and sensitivity to neurodegeneration when compared to DTI measurements.

Our study aimed at investigating whether both classical statistics and machine learning would allow the detection of GM differences between PPMS and RRMS, relying on 3D‐SHORE microstructural indices and taking DTI and conventional brain morphometry as benchmarks.

## Materials and Methods

### Participants

All patients gave their written informed consent prior to participating in the study. All procedures were performed in accordance with the Declaration of Helsinki (2008) and the study protocol was approved by the local Ethical Committee. The study population included two groups of MS patients (45 PPMS and 45 RRMS) recruited in our center. Inclusion criteria were: diagnosis of PPMS or RRMS based on McDonald 2010 diagnostic criteria^23^ and the availability of a standard anatomical 3T MRI scan acquired near the most recent neurological examination for excluding the concomitant presence of any other brain condition. Exclusion criteria were any condition that prevented the execution of an MRI acquisition, in particular: presence of any metal implant or objects known to be non‐compatible with MRI, such as pacemakers, medication pumps, aneurysm clips, metallic prosthesis, cochlear/retinal implants, hearing aids; claustrophobia; pregnancy. All imaging and clinical data were collected between the years 2015 and 2018. The physical disability status of each participant was measured with the Expanded Disability Status Scale (EDSS).^24^ The detailed demographic and clinical data are reported in Table 1.

**TABLE 1:** Demographic and Clinical Variables of the Studied Populations

Variables	RRMS	PPMS	*P*‐Value
Female/male	32/13	26/21	0.053
Age (years)	42.8 ± 9.9 (21–61)	47.4 ± 10.9 (23–69)	0.040
Disease duration (years)	7.3 ± 6.2 (1–26)	12.1 ± 7.8 (1–32)	0.002
EDSS score	2.8 ± 1.2 (0–5)	4.7 ± 1.3 (2–7)	<0.001

Data are shown as mean ± SD and numbers in parentheses indicate the range.

RRMS = relapsing–remitting multiple sclerosis; PPMS = primary progressive multiple sclerosis; EDSS, Expanded Disability Status Scale (EDSS).

### MRI Data Acquisition

MRI data acquisition was performed on a 3T Philips Achieva scanner (Philips Medical Systems, Best, The Netherlands) equipped with an 8‐channel head receiver coil. The following sequences were included in the protocol: 1) 3D T1‐weighted fast field echo for structural images (T1‐w, repetition time [TR]/echo time [TE] = 8.1/3 msec, flip angle [FA] = 8°, field of view [FOV] = 240 mm × 240 mm, 1 mm isotropic resolution, 180 slices); 2) 3D turbo spin echo T2‐w for structural images (TR/TE = 2500/228 msec, FA = 90°, FOV = 256 mm × 256 mm, 1 mm isotropic resolution, 180 slices); 3) 3D fluid‐attenuated inversion recovery (FLAIR) acquisition (TR/TE = 8000/290 msec, TI = 2356 msec, FA = 90°, FOV = 256 mm × 256 mm, 0.9 mm × 0.9 mm × 0.5 mm resolution, 180 slices); 4) dMRI acquisition with multiple *b*‐values (TR/TE = 9300/109 msec, FA = 90°, FOV = 112 mm × 112 mm, 2 mm isotropic resolution, 62 slices, *b*‐values = 700/2000 s/mm^2^ with 32/64 gradient directions respectively and 7 b0 volumes).

### Image Processing

All dMRI data were initially preprocessed using the Tortoise DIFFPREP pipeline (https://tortoise.nibib.nih.gov/tortoise) including denoising, image re‐sampling, corrections for motion, eddy‐current, and EPI distortions. FSL software (https://fsl.fmrib.ox.ac.uk/fsl/fslwiki/) was then used for brain extraction and masking. In addition, the T1‐w image of each participant was rigidly registered to the mean b0 volume in order to estimate the transformation matrix. DTI and 3D‐SHORE models were fitted to the preprocessed data using DIPY (https://dipy.org/).

For each subject, the FLAIR images were rigidly registered to the T1‐w ones using the FSL's flirt tool. The FLAIR‐hyperintense lesions were automatically segmented and filled in the T1‐w images using the Lesion Prediction Algorithm (LPA) available in the Lesion Segmentation Toolbox (LST) for SPM12 (www.statistical-modelling.de/lst.html). This operation was performed to avoid biased morphometric measurements due to the lesioned tissue.^25^ Of note, LPA does not require to set any manual threshold, and it is currently the recommended option considering its fast processing and high sensitivity in lesion detection.^26^ Each individually filled T1‐w image was then imported in the FreeSurfer software (http://surfer.nmr.mgh.harvard.edu/, Harvard University, Boston, MA, USA) to perform a complete brain parcellation with 112 anatomical regions of interests (ROIs). A subset of these cortical and subcortical ROIs that were considered particularly relevant for the pathology were retained for further analyses based on the literature: thalamus, caudate, putamen, hippocampus, insula, precuneus, superior‐frontal gyrus, posterior cingulate cortex, lateral occipital cortex, lingual cortex, and pericalcarine.^6^, ^27^

### Feature Extraction

Eight microstructural indices were calculated for each subject. The fractional anisotropy (FA) and mean diffusivity (MD)^14^ were derived from the DTI model (fitted only to data acquired at *b*‐value = 700 s/mm^2^), while generalized fractional anisotropy (GFA), propagator anisotropy (PA), mean square displacement (MSD), return to the origin/axes/plane probability (RTOP, RTAP, and RTPP, respectively) were estimated from the 3D‐SHORE model.^13^, ^28^, ^29^ The 11 ROIs, indicated in the previous section, were used as masks to extract the regional microstructure values for each dMRI index, after having projected the Freesurfer parcellation in the dMRI native space using the previously estimated transformation matrices. Starting from these values, for each participant and ROI the following statistical moments were extracted: mean, median, mode, skewness, SD, and kurtosis.

Regarding morphometry, volume (subcortical ROIs), and thickness (cortical ROIs) were obtained by Freesurfer. All measures were averaged across the two hemispheres, and volume measures were also normalized by the total intracranial volume (eTIV) as estimated from the T1‐wimages in Freesurfer.

### Support Vector Machine Analysis

**FIGURE 1:**
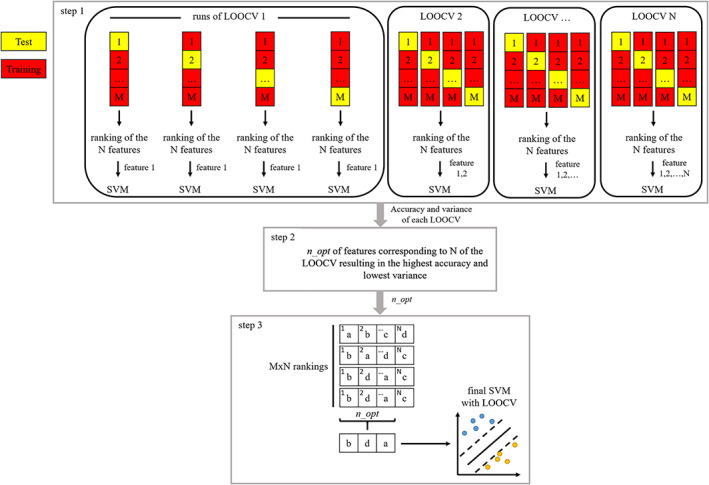
Representation of the three steps performed to obtain a feature selection and the final support vector machine (SVM) to classify primary progressive and relapsing–remitting multiple sclerosis patients, evaluated through leave one out cross validation (LOOCV). The variables *M* and *N* correspond to the number of subjects (90) and the total number of features explored in this study (539), respectively.

A complementary analysis was performed relying on a linear support vector machine (SVM) classifier with the twofold goal of identifying the most eloquent features and performing the two‐class classification task based on those.^30^ The underlying assumption is that consistency across methods in feature ranking would provide evidence of the robustness of the outcomes. To this end, a three‐step procedure was followed. In the first step, the optimal number of features, *n_opt*, was determined based on leave one out cross validation (LOOCV). In the second step, the *n_opt* features were selected by a ranking consensus criterion across all the tested models (539 × 90), and in the third step the resulting feature set was used to feed a SVM to check their discriminative power on the overall classification problem. An illustration of the three steps is reported in Fig. 1 and more details are provided hereafter.

**Step 1**—In order to choose the optimal number of features, LOOCV was repeated 539 times, corresponding to the total number of features, including both dMRI and morphometric descriptors.^11^ Using a progressively increasing number of features *n* with unit step, *n* = 1, 2, …, 539 (i.e., one feature was added at a time), for each run of the LOOCV the Feature Selection Code Library^31^ was applied to calculate the Fisher scores of all the 539 features. The first *n* features of the resulting ranking were then used to train and validate a linear SVM with standardization of the observations, sequential minimal optimization as solver, and cost 0.4662. This step resulted in a feature ranking for each of the 539 × 90 tested models, as well as the mean and the variance of the accuracy values for each *n*. The optimal cost was chosen by searching for the best accuracy reached by applying a linear SVM fitting with LOOCV, where the cost varied in a space defined by a vector of 100 evenly spaced points between 0.0005 and 512.

**Step 2**—The optimal number of features *n_opt* was obtained by looking for both the highest possible accuracy and the lowest possible variance. More in detail, the best accuracy and variance were first initialized as the ones obtained for *n* = 1, and updated when a higher or equal accuracy and a lower or equal variance were found for increasing values of *n*.

**Step 3**—The most occurring features in the first *n_opt* positions across all the model rankings were retained for feeding the final SVM. This was evaluated through LOOCV, and the corresponding confusion matrix along with accuracy, sensitivity, specificity, and precision indicators were calculated.

### Statistical Tests

Differences between the two MS phenotypes (RRMS, PPMS) were tested for age, disease duration, and EDSS distributions using an unpaired two‐sample *t*‐test, while a chi‐square test was performed to check for gender distribution differences.

Differences between the extracted dMRI features (the 6 distribution moments times the number of ROIs, resulting in 66 features) were evaluated by performing an analysis of covariance (ANCOVA), separately for each microstructural index. In particular, the MS disease staging (DISEASE), ROI, and distribution central and non‐central moments of different orders (FEAT) were used as factors, while age, disease duration, and EDSS were used as covariates. Post‐ hoc tests adjusted for multiple comparisons with Bonferroni correction were computed for the significant interactions. Accordingly, the morphometry information was analyzed with a two‐way ANCOVA using only DISEASE and ROI as factors, and age, disease duration, and EDSS as covariates. Also in this case, the significant interactions were further investigated with adjusted post hoc tests (Bonferroni). For all statistical tests, *P* < 0.05 was chosen as the threshold for significance.

## Results

### Group Comparison—Demographic and Clinical Information

Significant differences between the RRMS and PPMS groups were present in terms of age, disease duration, and EDSS. In particular, the PPMS group revealed significantly higher age, disease duration, and EDSS scores compared to RRMS. Conversely, gender distributions were not significantly different. A summary of these results is reported in Table 1.

### Group Comparison—dMRI Indices

**FIGURE 2:**
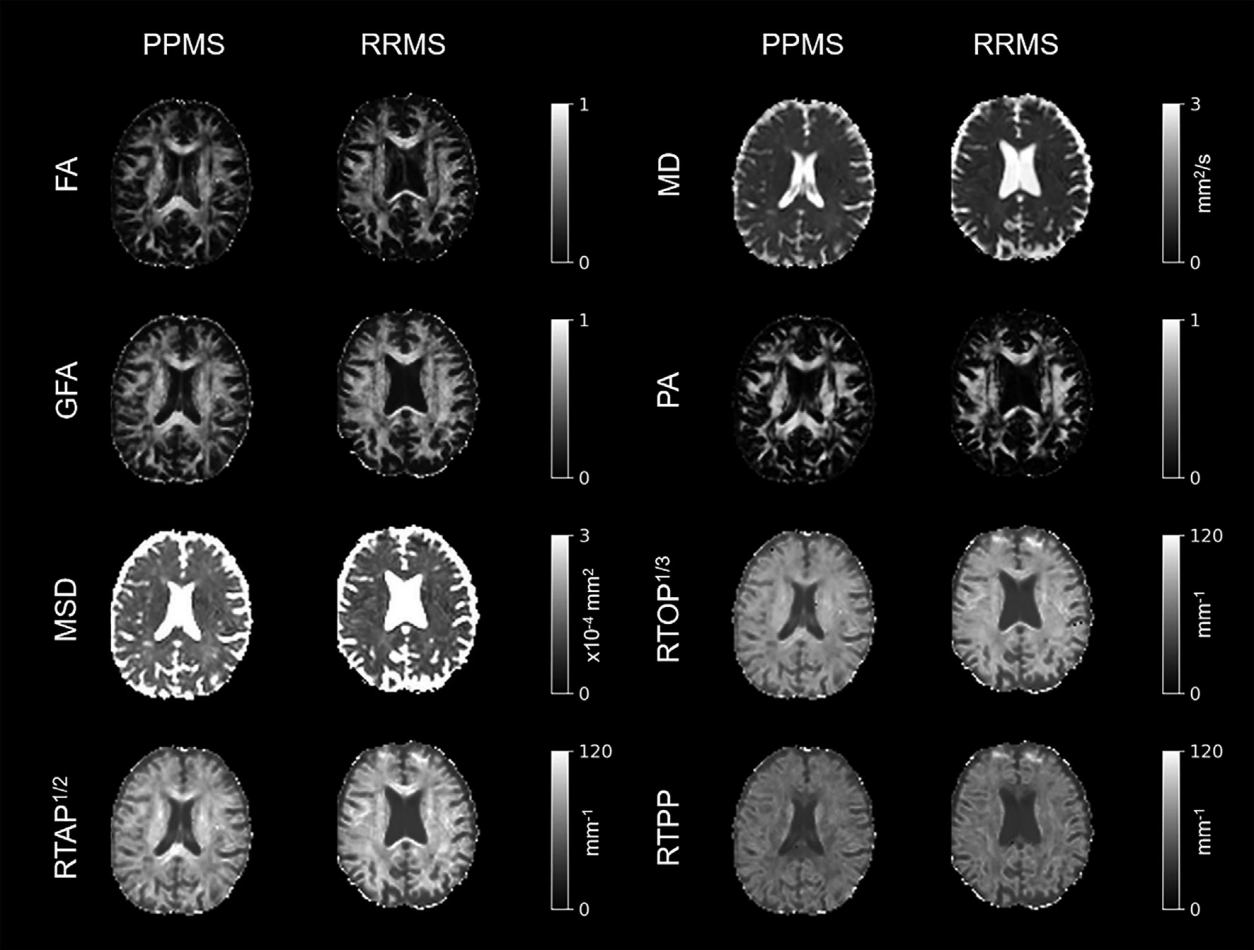
Microstructural indices from diffusion MRI (dMRI). For ease of comparison across RTOP, RTAP, and RTPP maps, the cubic‐ and square‐root of RTOP and RTAP were displayed. Axial slices of two representative patients (one primary progressive and one relapsing–remitting multiple sclerosis [PPMS and RRMS]) are reported for all microstructural indices. Images are displayed in radiological convention. FA = fractional anisotropy; MD = mean diffusivity; GFA = generalized fractional anisotropy; PA = propagator anisotropy; MSD = mean square displacement; RTOP = return to the origin probability; RTAP = return to the axis probability; RTPP = return to the plane probability.

The microstructural indices derived from the DTI and 3D‐SHORE models for two representative patients, one per group, are illustrated in Fig. 2. MD and MSD were hyperintense in areas of unrestricted diffusion, such as the cerebrospinal fluid, where all the other six indices were hypointense. High values in FA, GFA, and PA (bright areas) can be observed in regions where diffusion mostly happens along one preferred direction, such as the corpus callosum, and show the same type of contrast as RTPP, RTAP, and RTOP, though to a different extent.

**FIGURE 3:**
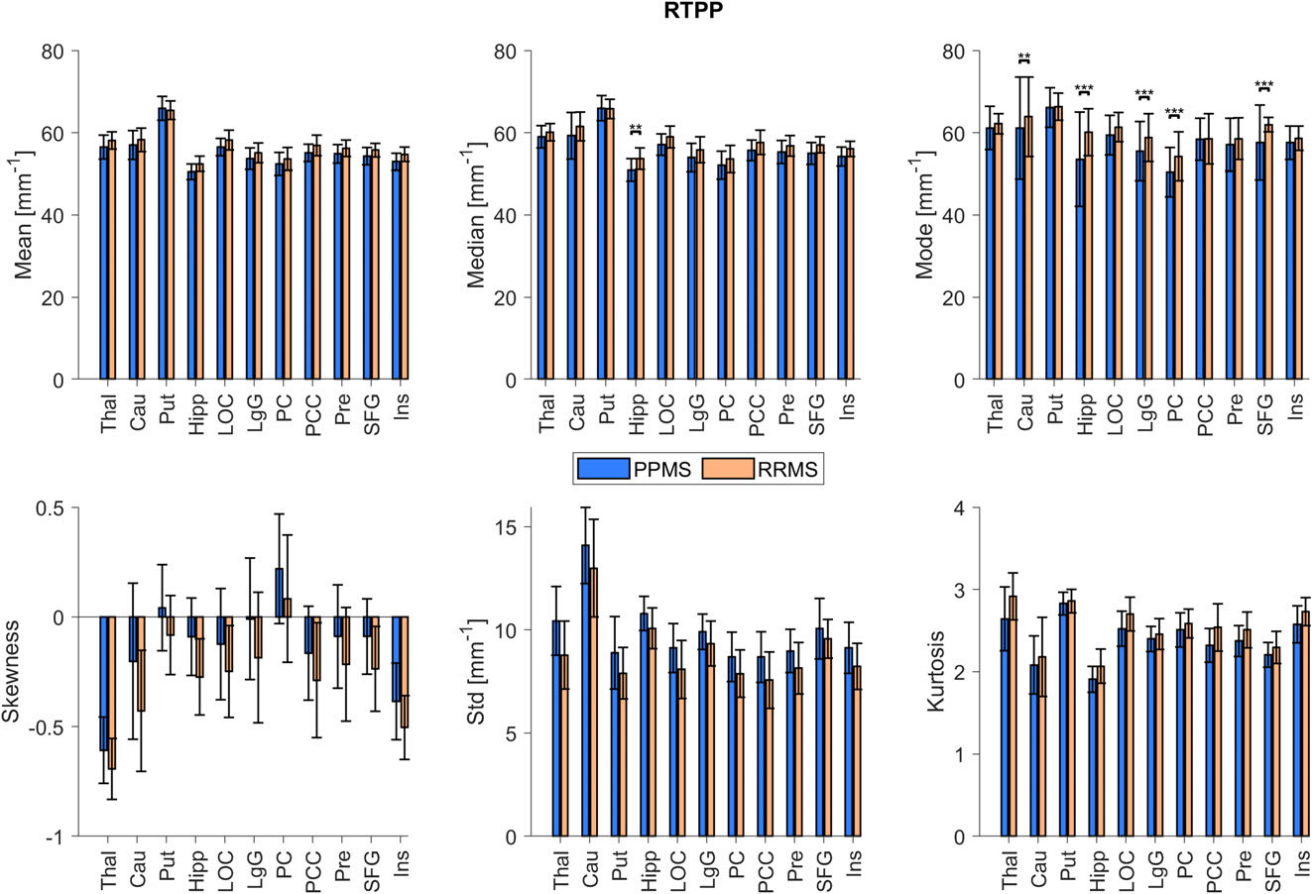
Return to the plane probability (RTPP) features for primary progressive and relapsing–remitting multiple sclerosis (PPMS and RRMS). For each region, mean, median, mode, skewness, SD, and kurtosis are reported as mean ± SD values across subjects (** *P*_Bonf_ < 0.01, *** *P_Bonf_* < 0.001). Thal = thalamus; Cau = caudate; Put = putamen; Hipp = hippocampus; LOC = lateral occipital cortex; LgG = lingual gyrus; PC = pericalcarine; PCC = posterior cingulate cortex; Pre = precuneus; SFG = superior frontal gyrus; Ins = insula.

A significant three‐way interaction (DISEASE*ROI*FEAT) was found for all the microstructural indices. Several significant between‐group differences, as shown in Fig. 3, were noted for the RTPP index (see Figs. S1–S7 in the Supplemental Material for the other indices). In particular, RTPP was different in two measures of centrality that are median and mode. In detail, the hippocampus was the only ROI whose RTPP‐median value for PPMS group was significantly lower for RRMS ones. Regarding RTPP‐mode, PPMS values were significantly lower than RRMS ones when considering the ROIs where this measure was significantly different between groups (including the hippocampus). Observing all the microstructural indices of the study, mode was significantly different between MS stages exclusively in the indices of restriction (RTOP, RTAP, and RTPP), while only kurtosis was found to be discriminative for all the other dMRI measurements. Exceptions were reported for the thalamus which also exhibited a significant between‐group difference for MD‐ and MSD‐skewness, and RTAP‐SD. We further noticed that, for all dMRI indices, the subcortical structures (thalamus, caudate, putamen, and hippocampus) were more frequently included among the ROIs highlighting any significant difference across groups.

### Group Comparison—Morphometric Descriptors

**FIGURE 4:**
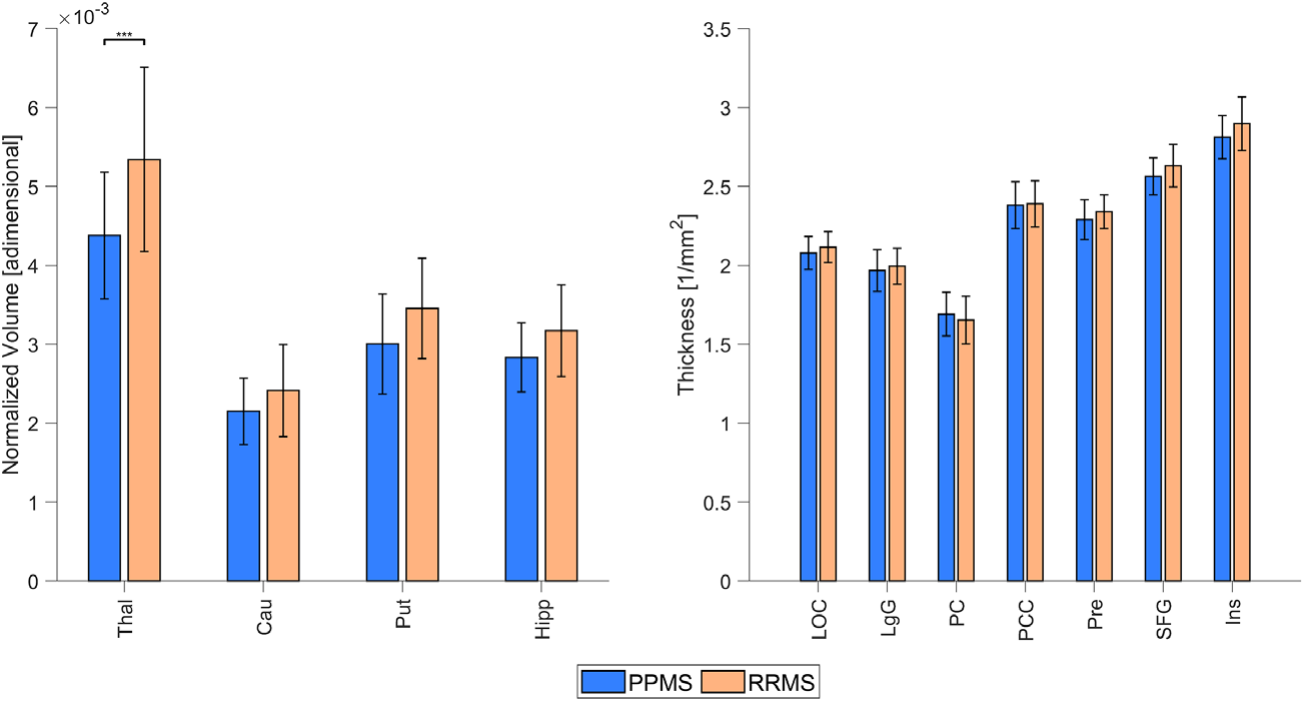
Morphometric measures for primary progressive and relapsing–remitting multiple sclerosis (PPMS and RRMS). Volume (left) and thickness (right) are reported as mean ± SD values across subjects (***P_Bonf_ < 0.001). Thal = thalamus; Cau = caudate; Put = putamen; Hipp = hippocampus; LOC = lateral occipital cortex; LgG = lingual gyrus; PC = pericalcarine; PCC = posterior cingulate cortex; Pre = precuneus; SFG = superior frontal gyrus; Ins = insula.

In terms of regional morphometric differences, a significant two‐way interaction (DISEASE*ROI) was detected for both volume and thickness values. As evidenced in Fig. 4, the thalamus was the only ROI featuring a significant difference between the two MS phenotypes, with PPMS group values significantly lower than RRMS ones. Conversely, no thickness values were deemed as statistically significant after multiple comparison correction (pericalcarine: *P*_Bonf_ = 0.109; superior‐frontal gyrus: *P*_Bonf_ = 0.409; lingual cortex: *P*_Bonf_ = 0.437; insula, precuneus, posterior cingular cortex, lateral occipital cortex: *P*_Bonf_ = 1.000).

### SVM

SVM analysis resulted in 12 features among which 10 corresponded to the hippocampus, while 2 were related to the thalamus. The selected features corresponding to the hippocampus were MSD‐ and MD‐SD, MSD‐ and MD‐mean, RTOP‐mode, RTPP‐skewness, RTOP‐, RTAP‐ and RTPP‐median, and RTPP‐mean. The selected features corresponding to the thalamus were RTAP‐ and RTPP‐SD.

**FIGURE 5:**
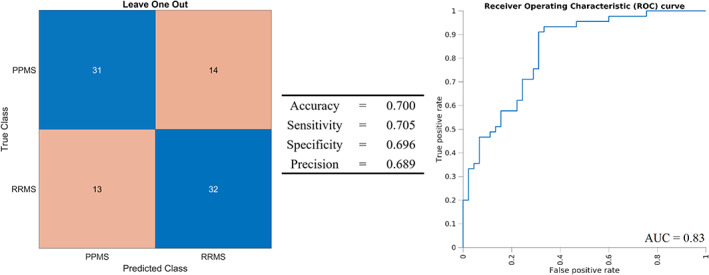
Performance of the leave one out cross validation evaluating the linear support vector machine using the features resulting from Fisher score based selection as predictors for classifying primary progressive vs. relapsing–remitting multiple sclerosis (PPMS and RRMS, respectively) patients. The confusion matrix (left) and related performance indicators (middle) are shown, alongside the corresponding receiver operating characteristic (ROC) curve (right) and the area under the curve (AUC) value.

Figure 5 summarizes the performance of the SVM, alongside the corresponding receiver operating characteristic curve (area under the curve = 0.83). The values of the confusion matrix demonstrated no particular imbalance in classifying one class with respect to the other (31 correctly classified PPMS vs. 32 correctly classified RRMS).

## Discussion

We investigated whether classical DTI and 3D‐SHORE microstructural descriptors were able to depict differences between PPMS and RRMS patients in a set of GM regions, as compared with standard morphometric measures. This intra‐pathology investigation is particularly challenging as subtle differences between highly similar descriptors need to be captured when missing a cohort of matched healthy control subjects. Our findings revealed that differential GM microstructure alterations between PPMS and RRMS can be detected using quantitative multiparametric MRI. This was particularly evident for dMRI‐derived indices, especially for the restriction ones (RTAP, RTOP, RTPP) which reported significantly different mode and median values.

The link between the observed dMRI measures and the underlying microstructural biophysical properties is not straightforward, mainly due to the inherent limitations in sensitivity due to the acquisition scheme,^7^ which makes the interpretation of these results particularly challenging. For this reason, the analysis of the distributions of the microstructural measures can better inform on the tissue modulations since it offers a more complete picture of the underlying process with respect to analyzing solely the mean value which is more sensitive to the presence of outliers.

### Microstructural Properties Distinguish PPMS from RRMS

While probing GM microstructural differences between PPMS and RRMS with dMRI, we used the classical DTI and the 3D‐SHORE models. The exploitation of 3D‐SHORE based measures in clinical studies has been proven to provide a detailed and specific tissue characterization, allowing to disentangle different conditions where DTI indices take the same values.^12^, ^13^ For instance, DTI cannot distinguish whether a reduction of FA is caused by crossing fibers or by a decrease of neural density in a voxel. Conversely, the joint exploitation of RTAP and RTPP can allow disentangling such ambiguity, as RTAP and RTPP both diminish in the case of neuronal density reduction, while RTAP decreases and RTPP increases for crossing fibers.^12^ Moreover, a decrease in anisotropy could indicate the presence of neuroinflammation or loss of myelination and neuronal loss. Therefore, irrespectively of the exact interpretation of the biophysical meaning of such indices, what it is relevant in the context is that they can capture the modulations of the tissue microstructural properties that subtend the two phenotypes of the pathology. The presence of GM microstructural abnormalities is in line with previous literature findings showing several microstructural alterations, in terms of either diffusivity, anisotropy, volume fraction, or mean kurtosis in lesion and normal‐appearing GM tissues in MS patients.^21^, ^22^ In particular, dMRI‐based indices have proven to detect earlier focal cortical pathological changes and provide a better assessment of the microstructural integrity, further highlighting the clinical value of cortical imaging, in line with recent recommendations.^32^

Investigating the dMRI measurement distributions statistics allowed to observe group differences otherwise not visible by using only the mean value. Median and mode are direct measures of the microstructural indices and they were significantly different between PPMS and RRMS only for descriptors of restriction, and more precisely RTPP. Among the ROIs, hippocampus appeared to be a key region showing group microstructure variations in both median and mode. The significant decrease in RTPP in PPMS in this region might be related to lower restriction to diffusion or reduction in neuronal density in hippocampus,^12^, ^17^ which was recently shown to be a significant feature in MS.^33^ Moreover, a recent study highlighted the early and central role of microstructural alterations in hippocampus as the first region undergoing microstructural changes. In addition, this study predicted significant hippocampal tissue loss after 1‐year of follow‐up in patients with clinically isolated syndrome, the premorbid phase of MS.^34^ Similarly, Rocca et al^35^ highlighted the importance of this region and suggested that the hippocampus might be one of the main potential targets for treatment. Rocca et al based their hypothesis on previous evidence demonstrating the hippocampus involvement in brain plasticity and neurogenesis, as in Ref. 36, as well as on several pathological studies that have shown regional alterations in terms of myelin, neuronal, and connectivity damage.

### Brain Morphometry Is Less Sensitive to PPMS and RRMS Differences Than dMRI

Different results were observed for the morphometric measures, as no significant group differences could be detected except for the thalamus, where PPMS volume values were significantly lower than RRMS ones. This agrees with previous studies suggesting that volume loss is a more prominent feature in PPMS, particularly in deep GM structures.^37^ On the other hand, several studies observed similar patterns of GM atrophy in MS subtypes.^6^, ^27^ In particular, Calabrese et al showed that, although the temporal evolution of cortical thinning differed among MS subtypes, insula, hippocampus, superior frontal gyrus, and cingulate cortex appeared atrophic in both RRMS and secondary progressive MS.^6^ Moreover, a recent study reported that thalamus, hippocampus, precuneus, and posterior cingulate cortex were affected early by atrophy in both PPMS and RRMS, whereas caudate and putamen showed late atrophy in PPMS and early atrophy in RRMS.^27^ Thus, these studies emphasized the consistent regional atrophy patterns in different presentations of MS appearing at the early or late phase of a given subtype. Our results agree with these findings, supporting the hypothesis that PPMS and RRMS might have common regional atrophy processes although the sequence of atrophy progression might depend on the disease duration.^27^

### Hippocampal Microstructural Properties Classify PPMS and RRMS

The relevance of dMRI‐derived indices in subcortical structures and especially in the hippocampus was confirmed by the features selected based on the Fisher score and the performance of the linear SVM. In fact, morphometric descriptors did not survive the selection process, and only RTAP‐ and RTPP‐SD in the thalamus were retained in addition to the other 10 features related to the hippocampus. Among these features, RTOP‐mode and RTPP‐median in the hippocampus, and RTAP‐SD in the thalamus also appeared as significantly different between the two MS stages in the statistical analysis. The SVM analysis thus provided additional evidence of the importance of the hippocampus region and the irrelevance of the anisotropy (FA, GFA, PA) and morphometric descriptors in favor of diffusivity (MD, MSD) and restriction (RTOP, RTAP, RTPP) ones. Several machine learning approaches have been applied in the current literature to distinguish MS from other pathologies or patients vs. healthy controls, generally relying on different MRI data and multi‐modal approaches.^38^ Among these, SVM is a classical and well‐established machine learning technique that has been widely used due to its simplicity and ease of use, associated with competitive performance. Thanks to these advantageous aspects, it is commonly used for benchmarking of more advanced/complex algorithms to provide evidence of the improvement of the costs/benefits (i.e., complexity vs. performance) trade‐off. In this study, we contrasted SVM to multivariate modeling with the twofold aim of providing first evidence of the suitability of machine learning methods for the problem at hand and to probe the persistency of the results across methods. Only a few studies have attempted to classify PPMS from RRMS using SVM with dMRI measures so far, exploiting global graph metrics derived from DTI‐based structural connectomes as predictors,^39^, ^40^ such as graph density, assortativity, and transitivity. In the first study,^39^ results obtained with unweighted connectivity graphs were considered more stable and less dependent on the pathological conditions with respect to those obtained with weighted ones. In view of this, the performance of our SVM was in line with the accuracy reached by Stamile et al in classifying PPMS and RRMS (accuracy = 68.3%), although using different features. Interestingly, Stamile et al classified more than two phenotypes (RRMS, PPMS, and secondary progressive MS) performing binary classifications and obtaining the highest variability for RRMS vs. PPMS. They hypothesized that the reason could be due to the substantial difference in the pathological processes underlying the PPMS and RRMS phenotypes. A wider analysis using several graph metrics was published in a second study,^40^ showing that a SVM having all these measures as predictors could reach a *F*‐measure = 0.706 (i.e., the weighted average of precision and recall) when distinguishing PPMS from RRMS. The performance of our SVM is in line with the values reported by these authors, although some differences in the study design should be emphasized, firstly the usage of measures derived from GM instead of WM, informing on the specific microstructural tissue properties instead on the global properties of the structural brain networks, and of a more complete set of dMRI indices rather than the classical DTI ones. Another important difference was in cardinality of the cohort. Indeed, 90 subjects balanced across the two phenotypes were analyzed in our work, while 24 RRMS and 17 PPMS were present in both the abovementioned studies. Finally, the SVM kernel was different (linear vs. graph^39^ and radial basis function kernels^40^).

Of note, the lack of a control group makes the classification task particularly challenging, as these two phenotypes might share some common patterns and features. However, efforts in this respect remain important especially for translational and precision medicine and for predicting the possible conversion to secondary progressive MS stage.

### Limitations

We acknowledge the limited sample size and the lack of a healthy control group as main limitations of the current study. Our cross‐sectional study design, involving two patient groups, is intrinsically more challenging to interpret compared to a patient vs. control group design since subtle differences are difficult to capture. The inclusion of a control group could help to reveal which ROIs are more affected by the pathology and guide further comparative analyses though not being informative of the differential tissue modulations between the two disease manifestations.

Moreover, in this study only two analytical models were considered for probing MS‐related GM microstructure damage, while many other models currently exist, such as NODDI and CHARMED, and could be similarly investigated for detecting MS‐related tissue alterations.

## Conclusion

Our study provides evidence for the higher sensitivity of dMRI in differentiating PPMS from RRMS based on regional GM microstructure properties compared to morphometry measures. Noteworthy, the GM region most sensitive to group differences was the hippocampus, suggesting a central role of this region in disease progression and calling for further investigation.

## Supporting information


**Fig. S1** Fractional Anisotropy (FA) features for primary progressive and relapsing‐remitting multiple sclerosis (PPMS and RRMS). For each region, mean, median, mode, skewness, standard deviation (std), and kurtosis are reported as mean ± standard deviation values across subjects (*P_Bonf_ < 0.05).
**Fig. S2** Mean Diffusivity (MD) features for primary progressive and relapsing‐remitting multiple sclerosis (PPMS and RRMS). For each region, mean, median, mode, skewness, standard deviation (std), and kurtosis are reported as mean ± standard deviation values across subjects (*P_Bonf_ < 0.05, ***P_Bonf_ < 0.001).
**Fig. S3** Generalized Fractional Anisotropy (GFA) features for primary progressive and relapsing‐remitting multiple sclerosis (PPMS and RRMS). For each region, mean, median, mode, skewness, standard deviation (std), and kurtosis are reported as mean ± standard deviation values across subjects (*P_Bonf_ < 0.05, **P_Bonf_ < 0.01, ***P_Bonf_ < 0.001).
**Fig. S4** Propagator Anisotropy (PA) features for primary progressive and relapsing‐remitting multiple sclerosis (PPMS and RRMS). For each region, mean, median, mode, skewness, standard deviation (std), and kurtosis are reported as mean ± standard deviation values across subjects (*P_Bonf_ < 0.05, ***P_Bonf_ < 0.001).
**Fig. S5** Mean Square Displacement (MSD) features for primary progressive and relapsing‐remitting multiple sclerosis (PPMS and RRMS). For each region, mean, median, mode, skewness, standard deviation (std), and kurtosis are reported as mean ± standard deviation values across subjects (**P_Bonf_ < 0.01, ***P_Bonf_ < 0.001).
**Fig. S6** Return To the Origin Probability (RTOP) features for primary progressive and relapsing‐remitting multiple sclerosis (PPMS and RRMS). For each region, mean, median, mode, skewness, standard deviation (std), and kurtosis are reported as mean ± standard deviation values across subjects (*P_Bonf_ < 0.05, **P_Bonf_ < 0.01, ***P_Bonf_ < 0.001).
**Fig. S7** Return To the Axis Probability (RTAP) features for primary progressive and relapsing‐remitting multiple sclerosis (PPMS and RRMS). For each region, mean, median, mode, skewness, standard deviation (std), and kurtosis are reported as mean ± standard deviation values across subjects (*P_Bonf_ < 0.05, **P_Bonf_ < 0.01, ***P_Bonf_ < 0.001).

## References

[1] Popescu BFG , Pirko I , Lucchinetti CF . Pathology of multiple sclerosis: Where do we stand? Continuum 2013;19(4):901‐921. 23917093 10.1212/01.CON.0000433291.23091.65 PMC3915566

[2] Lublin FD , Reingold SC , Cohen JA , et al. Defining the clinical course of multiple sclerosis: The 2013 revisions. Neurology 2014;83(3):278‐286. 24871874 10.1212/WNL.0000000000000560 PMC4117366

[3] Lucchinetti C , Brück W , Parisi J , et al. Heterogeneity of multiple sclerosis lesions: Implications for the pathogenesis of demyelination. Ann Neurol 2000;47(6):707‐717. 10852536 10.1002/1531-8249(200006)47:6<707::aid-ana3>3.0.co;2-q

[4] Huang WJ , Chen WW , Zhang X . Multiple sclerosis: Pathology, diagnosis and treatments. Exp Ther Med 2017;13(6):3163‐3166. 28588671 10.3892/etm.2017.4410 PMC5450788

[5] Cortese R , Collerone S , Ciccarelli O , Toosey A . Advances in brain imaging in multiple sclerosis. Ther Adv Neurol Disord 2019;12:1756286419859722. 31275430 10.1177/1756286419859722 PMC6598314

[6] Calabrese M , Reynolds R , Magliozzi R , et al. Regional distribution and evolution of gray matter damage in different populations of multiple sclerosis patients. PLoS One 2015;10(8):e0135428. 26267665 10.1371/journal.pone.0135428 PMC4534410

[7] Novikov DS , Fieremans E , Jespersen SN , et al. Quantifying brain microstructure with diffusion MRI: Theory and parameter estimation. NMR Biomed 2019;32(4):e3998. 30321478 10.1002/nbm.3998 PMC6481929

[8] Jelescu I , Budde M . Design and validation of diffusion MRI models of white matter. Front Phys 2017;28:61. 29755979 10.3389/fphy.2017.00061 PMC5947881

[9] Assaf Y , Basser PJ . Composite hindered and restricted model of diffusion (CHARMED) MR imaging of the human brain. Neuroimage 2005;27(1):48‐58. 15979342 10.1016/j.neuroimage.2005.03.042

[10] Zhang H , Schneider T , Wheeler‐Kingshott CA , et al. NODDI: Practical *in vivo* neurite orientation dispersion and density imaging of the human brain. Neuroimage 2012;61(4):1000‐1016. 22484410 10.1016/j.neuroimage.2012.03.072

[11] Lampinen B , Szczepankiewicz F , Mårtensson J , et al. Neurite density imaging versus imaging of microscopic anisotropy in diffusion MRI: A model comparison using spherical tensor encoding. Neuroimage 2017;147:517‐531. 27903438 10.1016/j.neuroimage.2016.11.053

[12] Zucchelli M , Brusini L , Méndez CA , et al. What lies beneath? Diffusion EAP‐based study of brain tissue microstructure. Med Image Anal 2016;32:145‐156. 27086167 10.1016/j.media.2016.03.008

[13] Özarslan E , Koay CG , Shepherd TM , et al. Mean apparent propagator (MAP) MRI: A novel diffusion imaging method for mapping tissue microstructure. Neuroimage 2013;78:16‐32. 23587694 10.1016/j.neuroimage.2013.04.016 PMC4059870

[14] Basser PJ , Mattiello J , LeBihan D . MR diffusion tensor spectroscopy and imaging. Biophys J 1994;66(1):259‐267. 8130344 10.1016/S0006-3495(94)80775-1 PMC1275686

[15] Avram AV , Sarlls JE , Barnett AS , et al. Clinical feasibility of using mean apparent propagator (MAP) MRI to characterize brain tissue microstructure. Neuroimage 2016;127:422‐434. 26584864 10.1016/j.neuroimage.2015.11.027 PMC4755846

[16] Brusini L , Obertino S , Boscolo Galazzo I , et al. Ensemble average propagator‐based detection of microstructural alterations after stroke. Int J Comput Assist Radiol Surg 2016;11(9):1585‐1597. 27368185 10.1007/s11548-016-1442-z

[17] Boscolo Galazzo I , Brusini L , Obertino S , et al. On the viability of diffusion MRI‐based microstructural biomarkers in ischemic stroke. Front Neurosci 2018;12:92. 29515362 10.3389/fnins.2018.00092 PMC5826355

[18] Brusini L , Cruciani F , Boscolo Galazzo I , et al. Multivariate data analysis suggests the link between brain microstructure and cognitive impairment in multiple sclerosis. In: Proceedings ‐ International Symposium on Biomedical Imaging, 2021, 685‐688.

[19] Calabrese M , Rinaldi F , Seppi D , et al. Cortical diffusion‐tensor imaging abnormalities in multiple sclerosis: A 3‐year longitudinal study. Radiology 2011;261(3):891‐898. 22031708 10.1148/radiol.11110195

[20] Woitek R , Leutmezer F , Dal‐Bianco A , et al. Diffusion tensor imaging of the normal‐appearing deep gray matter in primary and secondary progressive multiple sclerosis. Acta Radiol 2019;61(1):85‐92. 31169410 10.1177/0284185119852735

[21] Granberg T , Fan Q , Treaba CA , et al. In vivo characterization of cortical and white matter neuroaxonal pathology in early multiple sclerosis. Brain 2017;140(11):2912‐2926. 29053798 10.1093/brain/awx247 PMC5841207

[22] De Santis S , Bastiani M , Droby A , et al. Characterizing microstructural tissue properties in multiple sclerosis with diffusion MRI at 7 T and 3 T: The impact of the experimental design. Neuroscience 2019;403:17‐26. 29631021 10.1016/j.neuroscience.2018.03.048

[23] Polman CH , Reingold SC , Banwell B , et al. Diagnostic criteria for multiple sclerosis: 2010 revisions to the McDonald criteria. Ann Neurol 2011;69(2):292‐302. 21387374 10.1002/ana.22366 PMC3084507

[24] Kurtzke JH . Rating neurologic impairment in multiple sclerosis: An expanded disability status scale (EDSS). Neurology 1983;33(11):1444‐1452. 6685237 10.1212/wnl.33.11.1444

[25] Guo C , Ferreira D , Fink K , et al. Repeatability and reproducibility of FreeSurfer, FSL‐SIENAX and SPM brain volumetric measurements and the effect of lesion filling in multiple sclerosis. Eur Radiol 2019;29(3):1355‐1364. 30242503 10.1007/s00330-018-5710-x PMC6510869

[26] Schmidt P , Pongratz V , Küster P , et al. Automated segmentation of changes in FLAIR‐hyperintense white matter lesions in multiple sclerosis on serial magnetic resonance imaging. Neuroimage Clin 2019;23:101849. 31085465 10.1016/j.nicl.2019.101849 PMC6517532

[27] Eshaghi A , Marinescu RV , Young AL , et al. Progression of regional grey matter atrophy in multiple sclerosis. Brain 2018;141(6):1665‐1677. 29741648 10.1093/brain/awy088 PMC5995197

[28] Merlet SL , Deriche R . Continuous diffusion signal, EAP and ODF estimation via compressive sensing in diffusion MRI. Med Image Anal 2013;17(5):556‐572. 23602920 10.1016/j.media.2013.02.010

[29] Wu YC , Alexander AL . Hybrid diffusion imaging. Neuroimage 2007;36(3):617‐629. 17481920 10.1016/j.neuroimage.2007.02.050 PMC2428345

[30] Cortes C , Vapnik V . Support‐vector networks. Mach Learn 1995;20(3):273‐297.

[31] Roffo G , Melzi S , Castellani U , et al. Infinite latent feature selection: A probabilistic latent graph‐based ranking approach. In: Proceedings of ICCV, Venice; 2017, p 1407–1415.

[32] Filippi M , Rocca MA , Ciccarelli O , et al. MRI criteria for the diagnosis of multiple sclerosis: MAGNIMS consensus guidelines. Lancet Neurol 2016;15(3):292‐303. 26822746 10.1016/S1474-4422(15)00393-2 PMC4760851

[33] Carassiti D , Altmann DR , Petrova N , et al. Neuronal loss, demyelination and volume change in the multiple sclerosis neocortex. Neuropathol Appl Neurobiol 2018;44(4):377‐390. 28419506 10.1111/nan.12405

[34] Koubiyr I , Deloire M , Coupé P , et al. Differential gray matter vulnerability in the 1 year following a clinically isolated syndrome. Front Neurol 2018;9:824. 30364223 10.3389/fneur.2018.00824 PMC6193084

[35] Rocca MA , Barkhof F , De Luca J , et al. The hippocampus in multiple sclerosis. Lancet Neurol 2018;17(10):918‐926. 30264730 10.1016/S1474-4422(18)30309-0

[36] Kemperamm G , Song H , Gage FH . Neurogenesis in the adult hippocampus. Cold Spring Harb Perspect Biol 2015;7(9):a018812. 26330519 10.1101/cshperspect.a018812 PMC4563705

[37] Ceccarelli A , Rocca MA , Pagani E , et al. A voxel‐based morphometry study of grey matter loss in MS patients with different clinical phenotypes. Neuroimage 2008;42(1):315‐322. 18501636 10.1016/j.neuroimage.2008.04.173

[38] Zurita M , Montalba C , Labbé T , et al. Characterization of relapsing‐remitting multiple sclerosis patients using support vector machine classifications of functional and diffusion MRI data. Neuroimage Clin 2018;20:724‐730. 30238916 10.1016/j.nicl.2018.09.002 PMC6148733

[39] Stamile C , Kocevar G , Hannoun S , et al. A graph based classification method for multiple sclerosis clinical forms using support vector machine. In: Proceedings of the ICML Workshop on MLMMI, Lille; 2015, p 57–64.

[40] Kocevar G , Stamile C , Hannoun S , et al. Graph theory‐based brain connectivity for automatic classification of multiple sclerosis clinical courses. Front Neurosci 2016;10:478. 27826224 10.3389/fnins.2016.00478 PMC5078266

