# Supplementary material for: Unraveling the MRI‐Based Microstructural Signatures Behind Primary Progressive and Relapsing–Remitting Multiple Sclerosis Phenotypes
Source: J Magn Reson Imaging. 2021 Jun 30;55(1):154–63. doi: 10.1002/jmri.27806 (PMC9290631; doi:10.1002/jmri.27806)
Supplement: Supplementary file 1 — Fig. S1 Fractional Anisotropy (FA) features for primary progressive and relapsing‐remitting multiple sclerosis (PPMS and RRMS). For each region, mean, median, mode, skewness, standard deviation (std), and kurtosis are reported as mean ± standard deviation values across subjects (*P Bonf < 0.05). Fig. S2 Mean Diffusivity (MD) features for primary progressive and relapsing‐remitting multiple sclerosis (PPMS and RRMS). For each region, mean, median, mode, skewness, standard deviation (std), and kurtosis are reported as mean ± standard deviation values across subjects (*P Bonf < 0.05, ***P Bonf < 0.001). Fig. S3 Generalized Fractional Anisotropy (GFA) features for primary progressive and relapsing‐remitting multiple sclerosis (PPMS and RRMS). For each region, mean, median, mode, skewness, standard deviation (std), and kurtosis are reported as mean ± standard deviation values across subjects (*P Bonf < 0.05, **P Bonf < 0.01, ***P Bonf < 0.001). Fig. S4 Propagator Anisotropy (PA) features for primary progressive and relapsing‐remitting multiple sclerosis (PPMS and RRMS). For each region, mean, median, mode, skewness, standard deviation (std), and kurtosis are reported as mean ± standard deviation values across subjects (*P Bonf < 0.05, ***P Bonf < 0.001). Fig. S5 Mean Square Displacement (MSD) features for primary progressive and relapsing‐remitting multiple sclerosis (PPMS and RRMS). For each region, mean, median, mode, skewness, standard deviation (std), and kurtosis are reported as mean ± standard deviation values across subjects (**P Bonf < 0.01, ***P Bonf < 0.001). Fig. S6 Return To the Origin Probability (RTOP) features for primary progressive and relapsing‐remitting multiple sclerosis (PPMS and RRMS). For each region, mean, median, mode, skewness, standard deviation (std), and kurtosis are reported as mean ± standard deviation values across subjects (*P Bonf < 0.05, **P Bonf < 0.01, ***P Bonf < 0.001). Fig. S7 Return To the Axis Probability (RTAP) features for prim [file JMRI-55-154-s001.docx]

**
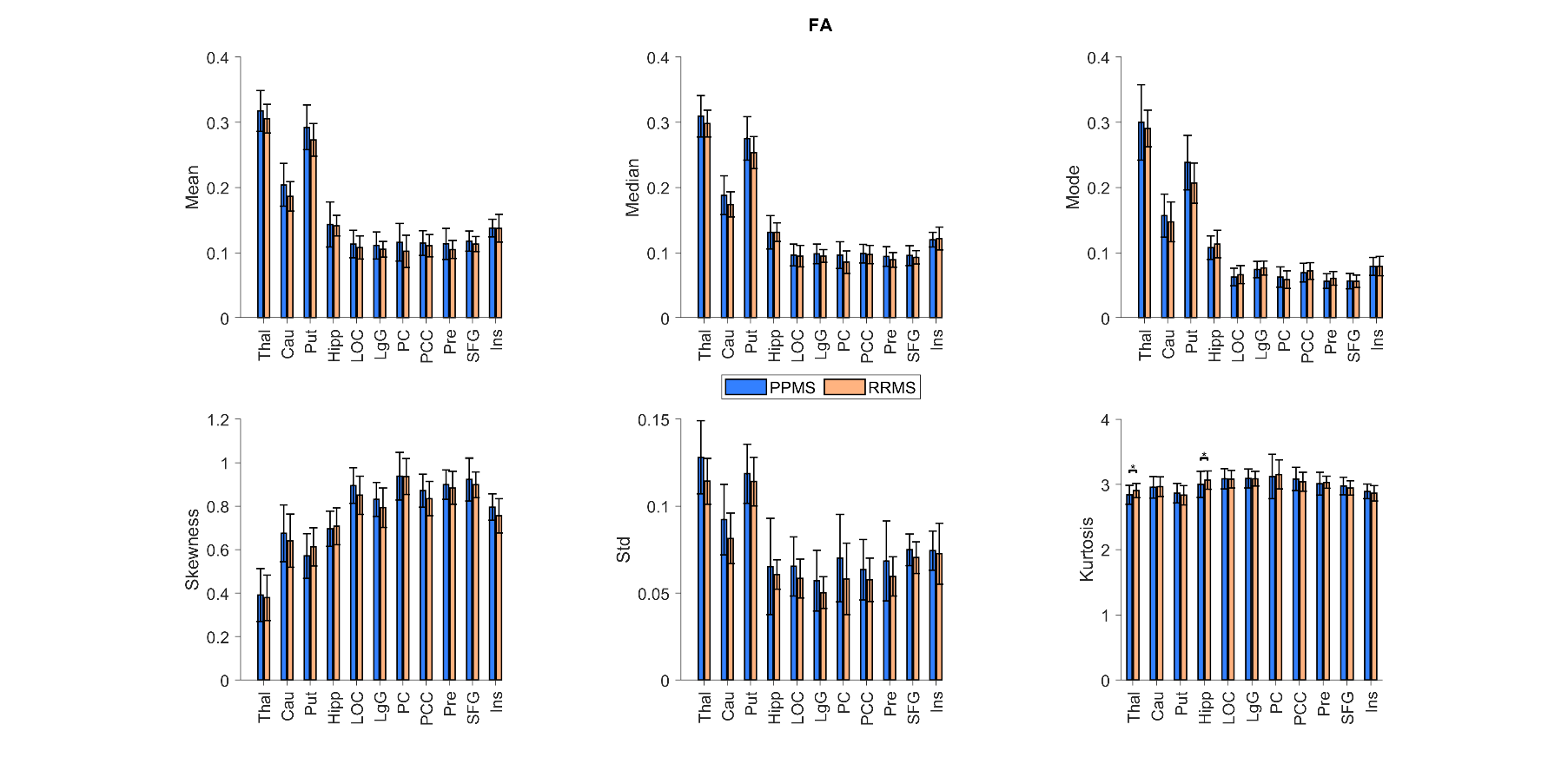
**

**Fig. S1** Fractional Anisotropy (FA) features for primary progressive and relapsing-remitting multiple sclerosis (PPMS and RRMS). For each region, mean, median, mode, skewness, standard deviation (std), and kurtosis are reported as mean ± standard deviation values across subjects (* *P*_Bonf_ < 0.05).

**
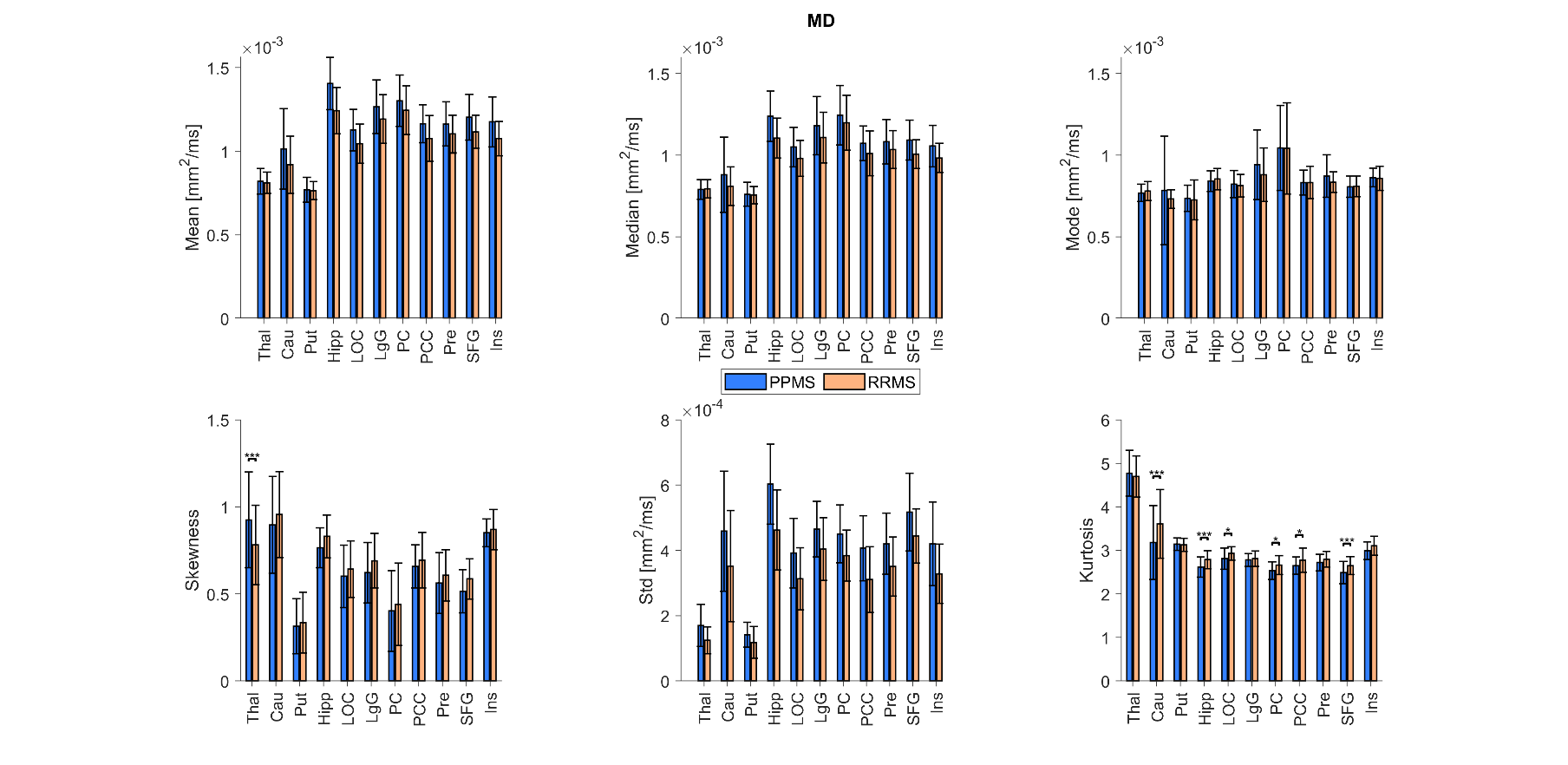
**

**Fig. S2** Mean Diffusivity (MD) features for primary progressive and relapsing-remitting multiple sclerosis (PPMS and RRMS). For each region, mean, median, mode, skewness, standard deviation (std), and kurtosis are reported as mean ± standard deviation values across subjects (* *P*_Bonf_ < 0.05, *** *P*_Bonf_ < 0.001).

**
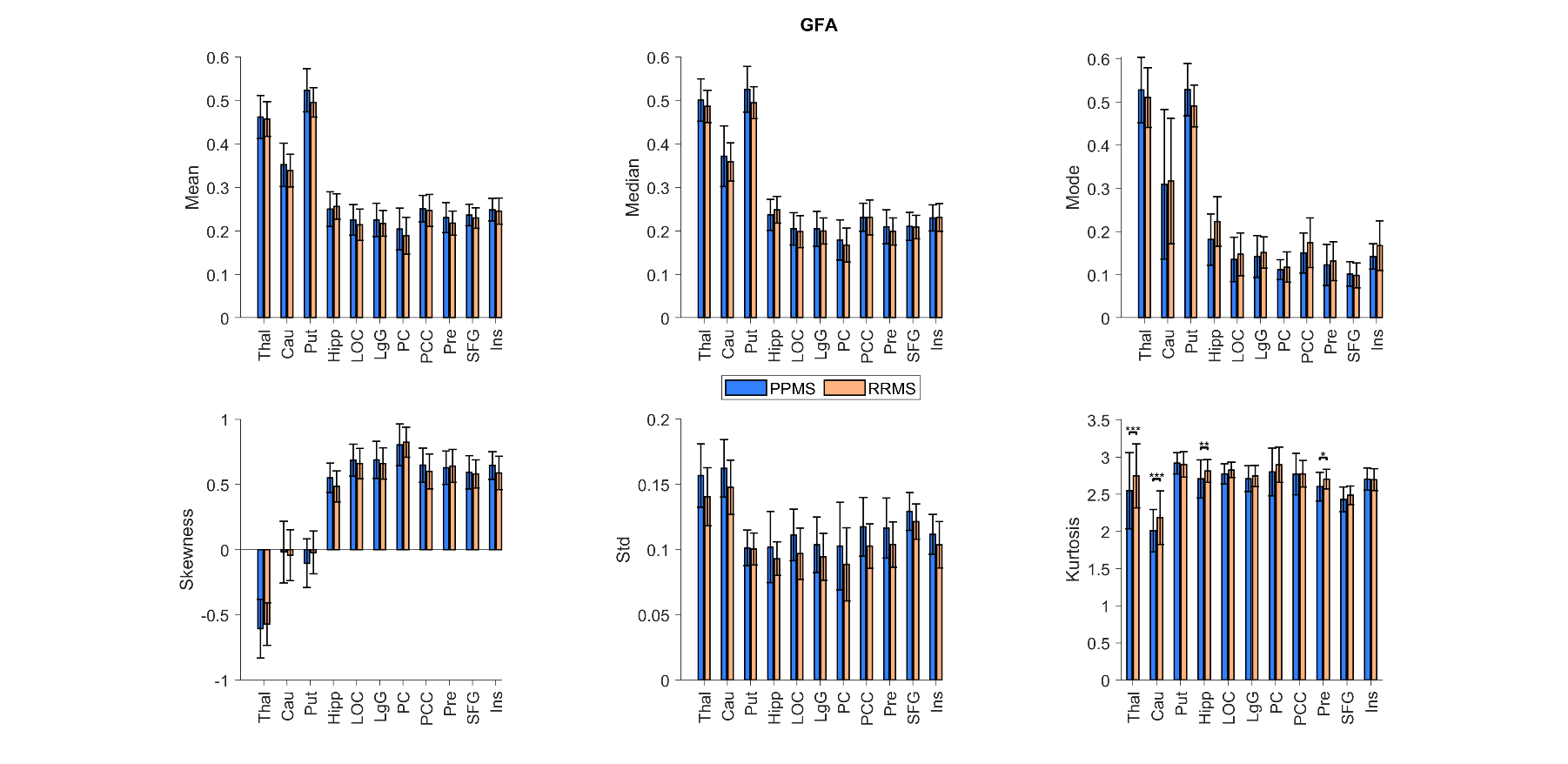
**

**Fig. S3** Generalized Fractional Anisotropy (GFA) features for primary progressive and relapsing-remitting multiple sclerosis (PPMS and RRMS). For each region, mean, median, mode, skewness, standard deviation (std), and kurtosis are reported as mean ± standard deviation values across subjects (* *P*_Bonf_ < 0.05, ** *P*_Bonf_ < 0.01, *** *P*_Bonf_ < 0.001).

**
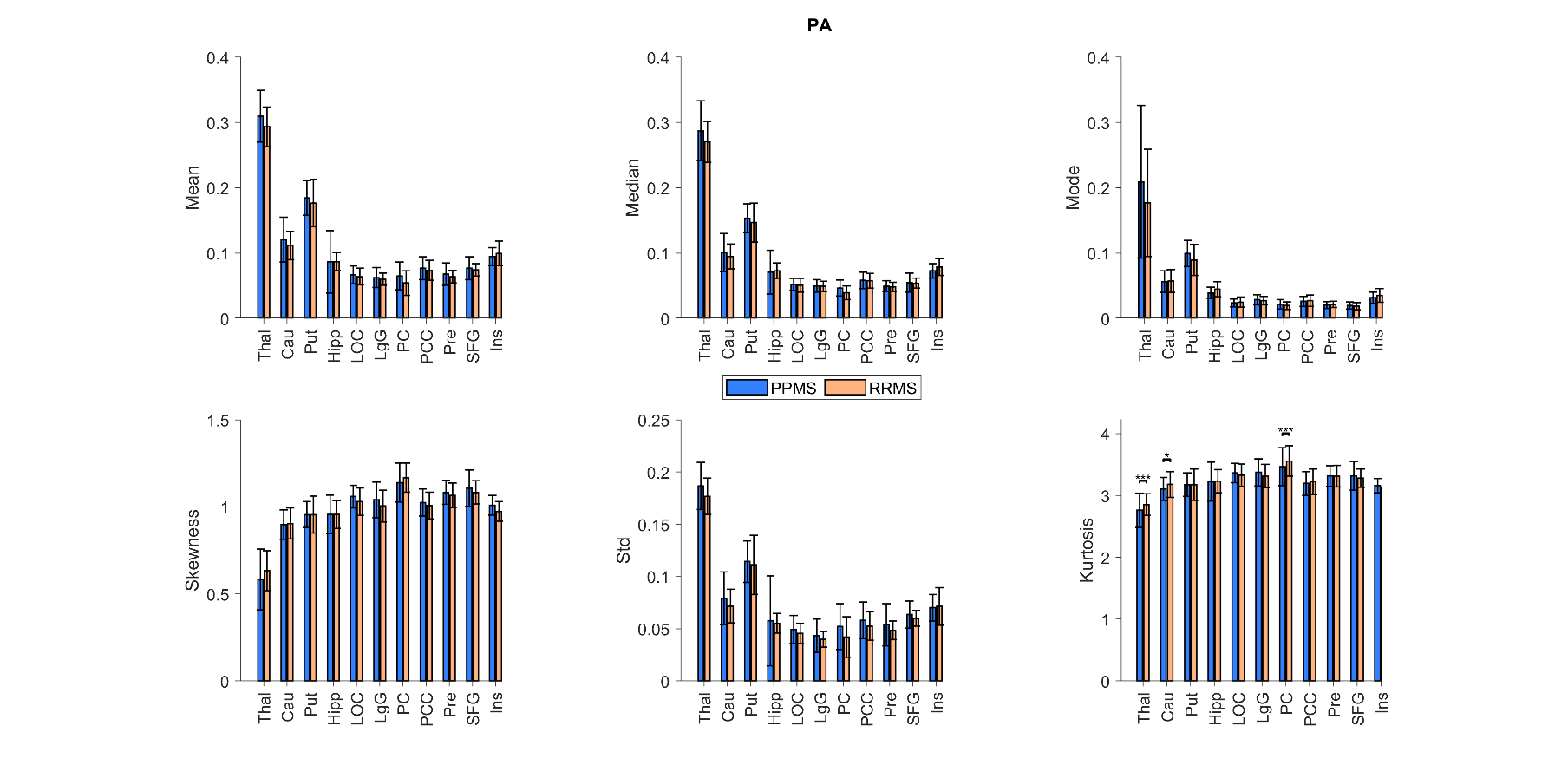
**

**Fig. S4** Propagator Anisotropy (PA) features for primary progressive and relapsing-remitting multiple sclerosis (PPMS and RRMS). For each region, mean, median, mode, skewness, standard deviation (std), and kurtosis are reported as mean ± standard deviation values across subjects (* *P*_Bonf_ < 0.05, *** *P*_Bonf_ < 0.001).

**
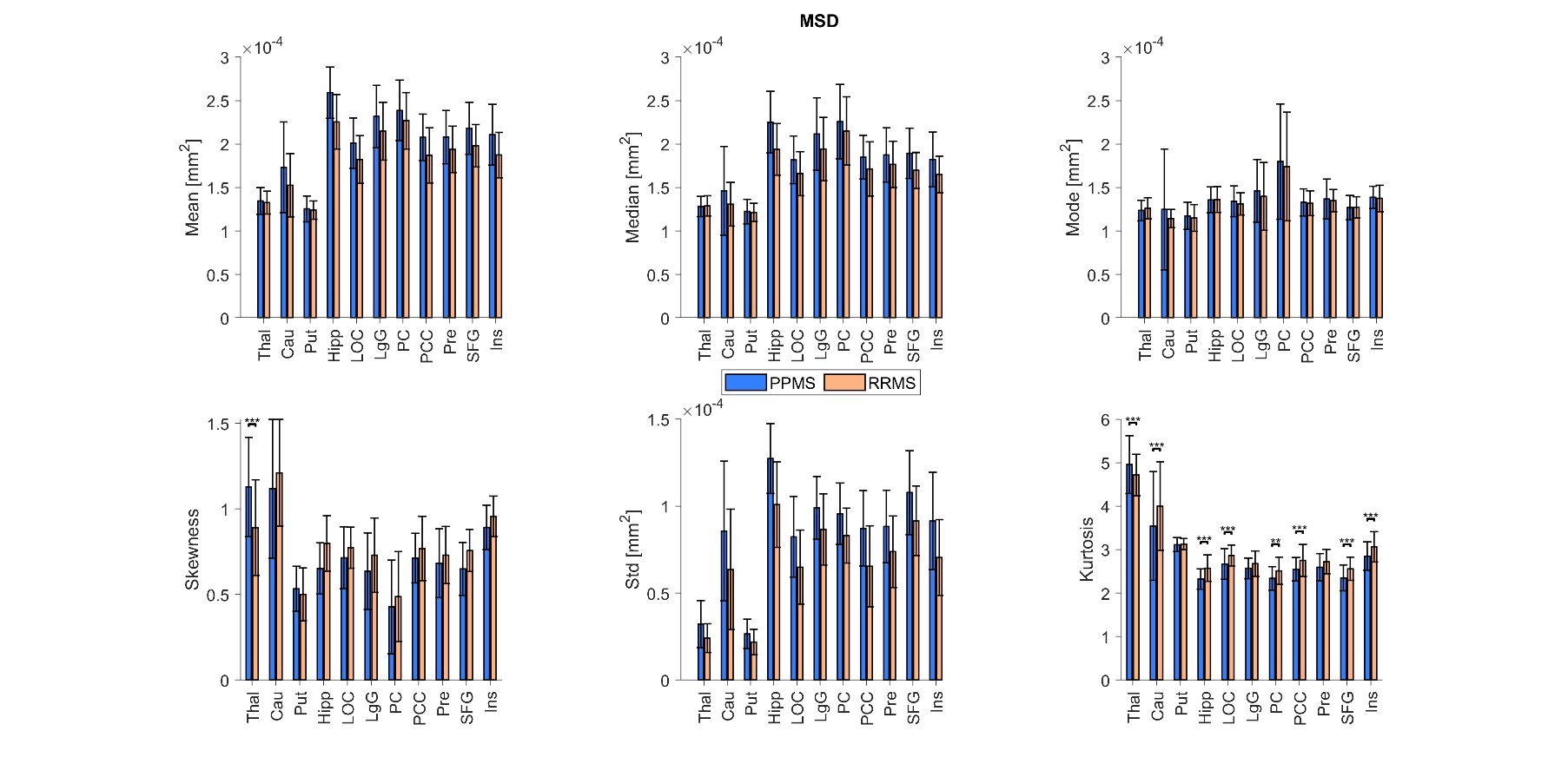
**

**Fig. S5** Mean Square Displacement (MSD) features for primary progressive and relapsing-remitting multiple sclerosis (PPMS and RRMS). For each region, mean, median, mode, skewness, standard deviation (std), and kurtosis are reported as mean ± standard deviation values across subjects (** *P*_Bonf_ < 0.01, *** *P*_Bonf_ < 0.001).

**
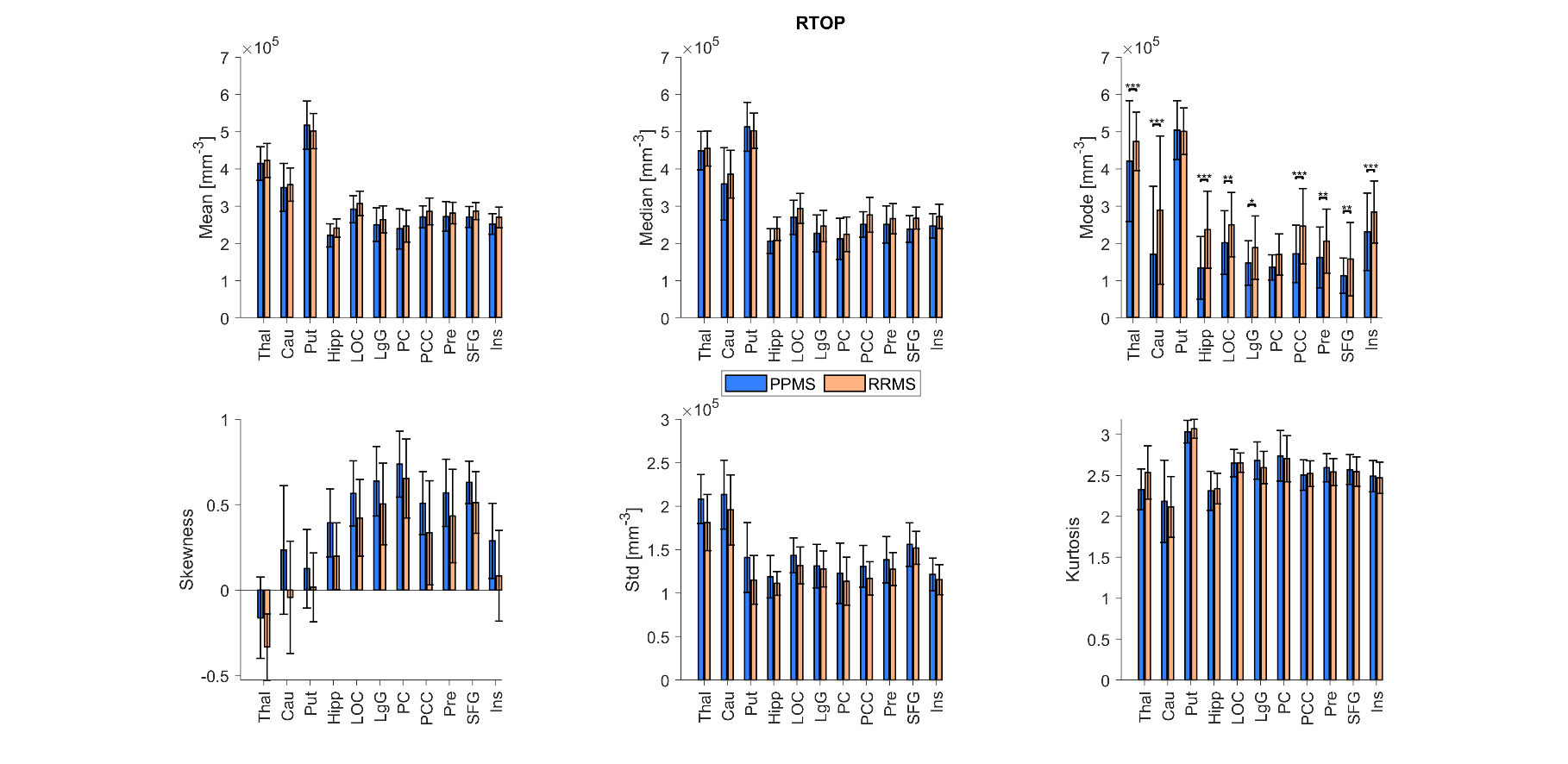
**

**Fig. S6** Return To the Origin Probability (RTOP) features for primary progressive and relapsing-remitting multiple sclerosis (PPMS and RRMS). For each region, mean, median, mode, skewness, standard deviation (std), and kurtosis are reported as mean ± standard deviation values across subjects (* *P*_Bonf_ < 0.05, ** *P*_Bonf_ < 0.01, *** *P*_Bonf_ < 0.001).

**
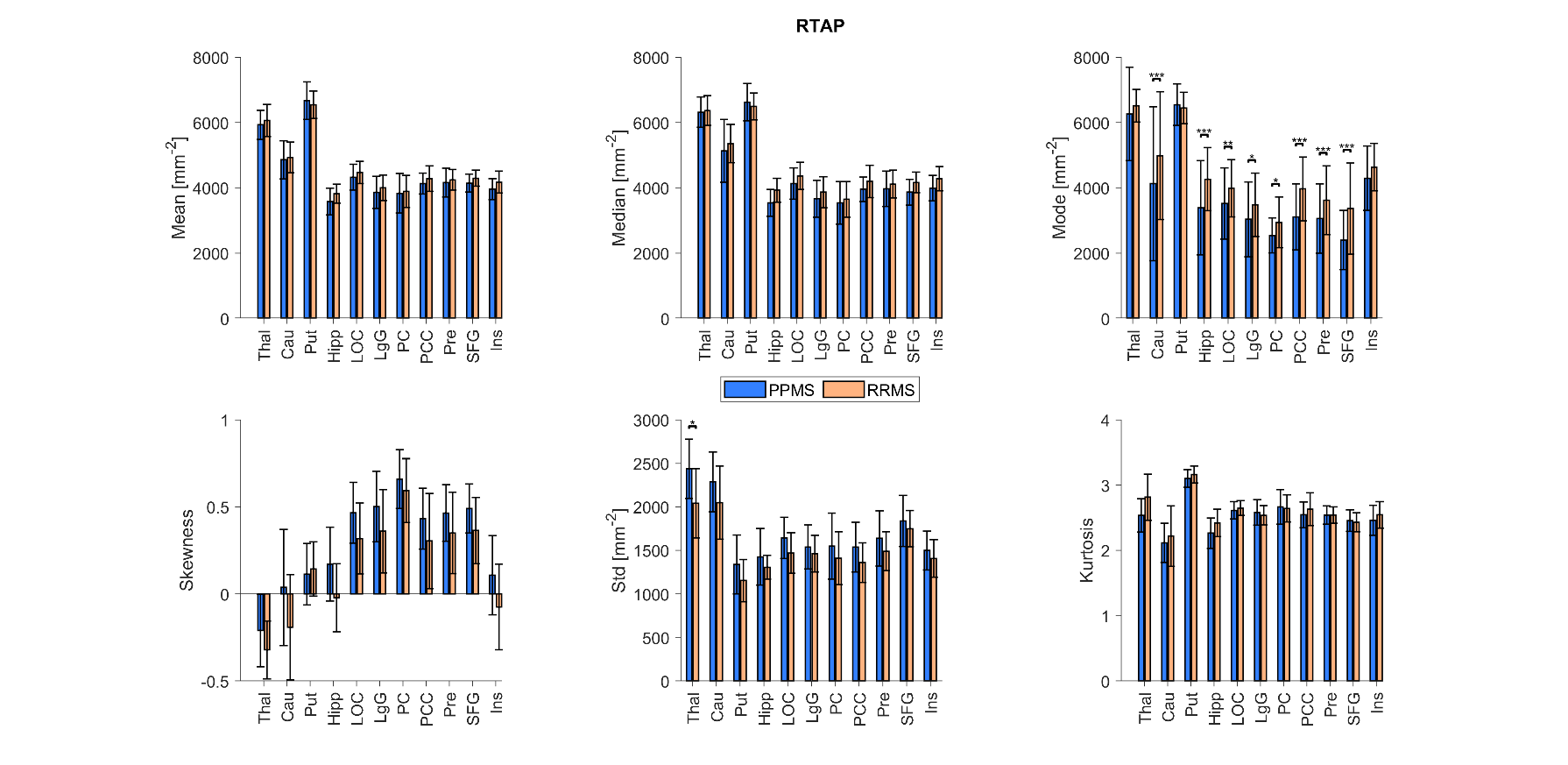
**

**Fig. S7** Return To the Axis Probability (RTAP) features for primary progressive and relapsing-remitting multiple sclerosis (PPMS and RRMS). For each region, mean, median, mode, skewness, standard deviation (std), and kurtosis are reported as mean ± standard deviation values across subjects (* *P*_Bonf_ < 0.05, ** *P*_Bonf_ < 0.01, *** *P*_Bonf_ < 0.001).
